# Phenotypic and genetic variation of ultraviolet–visible-infrared spectral wavelengths of bovine meat

**DOI:** 10.1038/s41598-021-93457-5

**Published:** 2021-07-06

**Authors:** Giovanni Bittante, Simone Savoia, Alessio Cecchinato, Sara Pegolo, Andrea Albera

**Affiliations:** 1 Department of Agronomy, Food, Natural Resources, Animals and Environment (DAFNAE), University of Padova (Padua), viale dell’Università 16, 35020 Legnaro, PD Italy; 2 Associazione Nazionale Allevatori Bovini di Razza Piemontese, Strada Trinità 32/A, 12061 Carrù, CN Italy; 3 Department of Animal Breeding and Genetics, Interbull Centre, SLU, PO Box 7023, 750 07 Uppsala, Sweden

**Keywords:** Biochemistry, Genetics

## Abstract

Spectroscopic predictions can be used for the genetic improvement of meat quality traits in cattle. No information is however available on the genetics of meat absorbance spectra. This research investigated the phenotypic variation and the heritability of meat absorbance spectra at individual wavelengths in the ultraviolet–visible and near-infrared region (UV–Vis-NIR) obtained with portable spectrometers. Five spectra per instrument were taken on the ribeye surface of 1185 Piemontese young bulls from 93 farms (13,182 Herd-Book pedigree relatives). Linear animal model analyses of 1481 single-wavelengths from UV–Vis-NIRS and 125 from Micro-NIRS were carried out separately. In the overlapping regions, the proportions of phenotypic variance explained by batch/date of slaughter (14 ± 6% and 17 ± 7%,), rearing farm (6 ± 2% and 5 ± 3%), and the residual variances (72 ± 10% and 72 ± 5%) were similar for the UV–Vis-NIRS and Micro-NIRS, but additive genetics (7 ± 2% and 4 ± 2%) and heritability (8.3 ± 2.3% vs 5.1 ± 0.6%) were greater with the Micro-NIRS. Heritability was much greater for the visible fraction (25.2 ± 11.4%), especially the violet, blue and green colors, than for the NIR fraction (5.0 ± 8.0%). These results allow a better understanding of the possibility of using the absorbance of visible and infrared wavelengths correlated with meat quality traits for the genetic improvement in beef cattle.

## Introduction

Spectroscopy of electro-magnetic radiations, especially in the range of near-infrared (NIRS) and visible (Vis) wavelengths, is a secondary methodology often used in analyses of food matrices^1,2^. In the case of meat, and particularly beef, chemometric approaches have been developed for predicting chemical characteristics, such as proximate analysis^3–5^, fatty acid profiles^6,7^, and mineral contents^8^, as well as physical characteristics, like meat color traits, drip and cooking meat losses, tenderness, and other technological and organoleptic properties^9,10^.

In the case of dairy species, spectroscopic predictions (especially Fourier transform near- and mid-infrared, FTIR) constitute the reference method at the population level for the genetic improvement of milk fat, protein and casein contents, and has been tested for improving the fatty acid profile^11,12^, the protein profile^13^, cheese-making properties^14,15^, as well as traits related to animal efficiency^16^, health and welfare^17,18^, and the environmental impact of the dairy chain^19,20^. This approach is favored for milk as samples are easy and inexpensive to collect, are homogeneous and representative, and can be taken periodically from all lactating females during their entire productive lives. This is not the case with meat production: meat can be sampled only once after slaughter of the animal, it is a highly heterogeneous material, and sampling can depreciate the whole carcass. Nevertheless, some research on the possible use of NIRS for genetically improving beef quality has been carried out^10,21^. Moreover, the practical obstacles to establishing a selection scheme for the genetic improvement of meat quality traits have recently been partially offset by the availability of small, portable and robust spectrometers^22^ that can collect spectra directly from the meat surface in the working environment (abattoirs, meat processing plants, retail outlets, etc.) without the need to take, transport, preserve and process samples^23^. Portable spectrometers differ greatly in size, spectrum extension and definition, technical features, and cost.

To expand knowledge in this field, we set up a large-scale study on the prediction of meat quality in Piemontese young bulls using portable NIR spectrometers (Qualipiem project). The first steps taken were: evaluation of 6 beef farming systems and other phenotypic sources of variation in carcass and meat quality traits using gold standard laboratory analyses^24^; estimation of their quantitative genetic parameters^25^ and genome-wide associations, and analyses of pathways^26^; prediction of meat quality traits from spectra collected on the muscle surface in the abattoir (hence without taking meat samples) using two spectrometers differing greatly in size, ease of use and cost^27^; estimation of the genetic parameters of meat quality traits predicted by the spectrometers compared with those obtained through gold-standard analyses^28^.

In using visible-infrared spectra for genetic improvement at the population level, it is clearly implicitly assumed that the absorbance of some wavelengths are heritable and correlated with the trait for selection, as was found for milk at the genetic^29,30^ and genomic levels^31^. In the case of meat species, to our knowledge, there is no information on the genetics of meat absorbance spectra.

The main objective of this study was therefore to investigate the variability—and particularly the heritability—of the absorbance spectra of beef in the range of the ultraviolet, visible and near-infrared radiations obtained with portable spectrometers. The specific aims were: to analyze, for each individual visible and near-infrared wavelength, the proportions of genetic and environmental (farm of origin, batch/date of slaughter, residual variation) causes of variation in absorbance; to calculate the heritability coefficient of absorbance at every individual wavelength; to compare, for genetic purposes, very different instruments (a heavy, expensive, top-of-the-range, broad-range, high-definition spectrometer, and an inexpensive, miniaturized, narrow-range, low-definition spectrometer).

## Results

**Figure 1:**
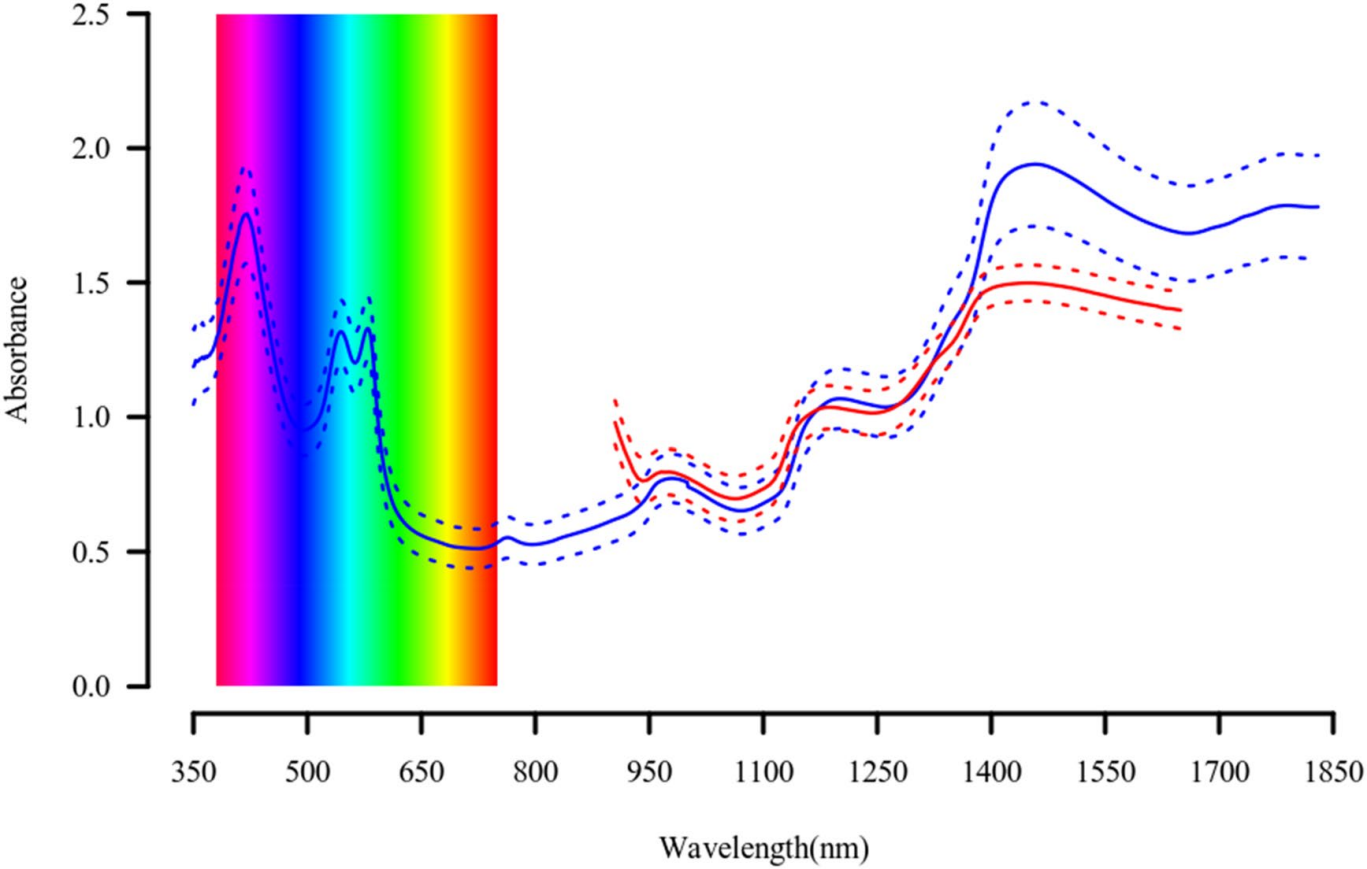
Variability of UV–Vis-NIRS and Micro-NIRS meat spectra. Average (solid line) and standard deviation interval (between dotted lines) of absorbance spectra of 5-6th rib cross-sectional area of *longissimus thoracis* muscle of Piemontese young bulls obtained using portable UV–Vis-NIRS (blue color) and Micro-NIRS (red color) instruments (the spectrum of each animal was obtained as average of 5 spectra taken in different sites of the muscle sectional area, each one with three replicates).

The average values and standard deviations of the absorbances recorded on the muscle surface by the two spectrometers in the fractions of the spectrum covered by both instruments were compared dividing the spectra in three portions (Fig. 1). Comparing the spectra of the two instruments in their common range (905–1650 nm), it is possible to see that the average absorbance of the 8 wavelengths recorded by the Micro-NIRS in the initial phase of its spectrum (905–950 nm) was higher and exhibited less variability than that of the corresponding 50 wavelengths recorded in the same interval by UV–Vis-NIRS (Fig. 1). The remaining common portions of each spectrum were similar to each other till 1400 nm (Fig. 1). The average absorbance and standard deviation of the 42 wavelengths recorded by the Micro-NIRS in last part of the spectrum (1400–1650 nm) were, in contrast, lower than those of the 250 wavelengths recorded in the same interval by the UV–Vis-NIRS (Fig. 1).

**Figure 2:**
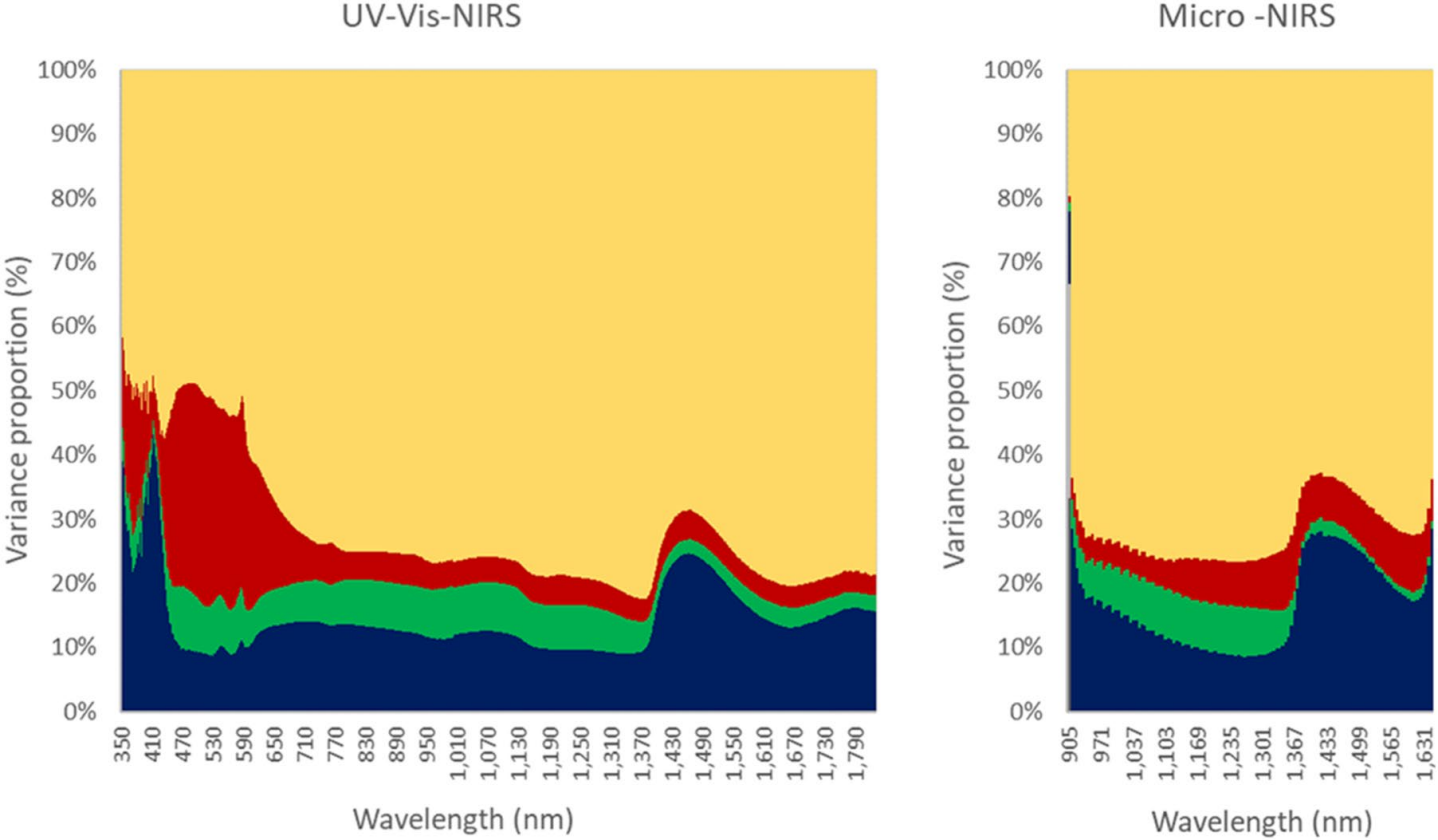
Proportions of the variances of the different sources of variation in absorbance. Proportion of variance due to batch (blue color), herd (green color), additive genetic (red color), and residual (yellow color) of absorbance of each individual wavelength of the spectra of Piemontese young bulls using portable UV–Vis-NIRS (left graph) and Micro-NIRS (right graph) spectrometers. Spectra taken on the 5–6th rib cross-sectional area of *longissimus thoracis* muscle the day after slaughter.

**Figure 3:**
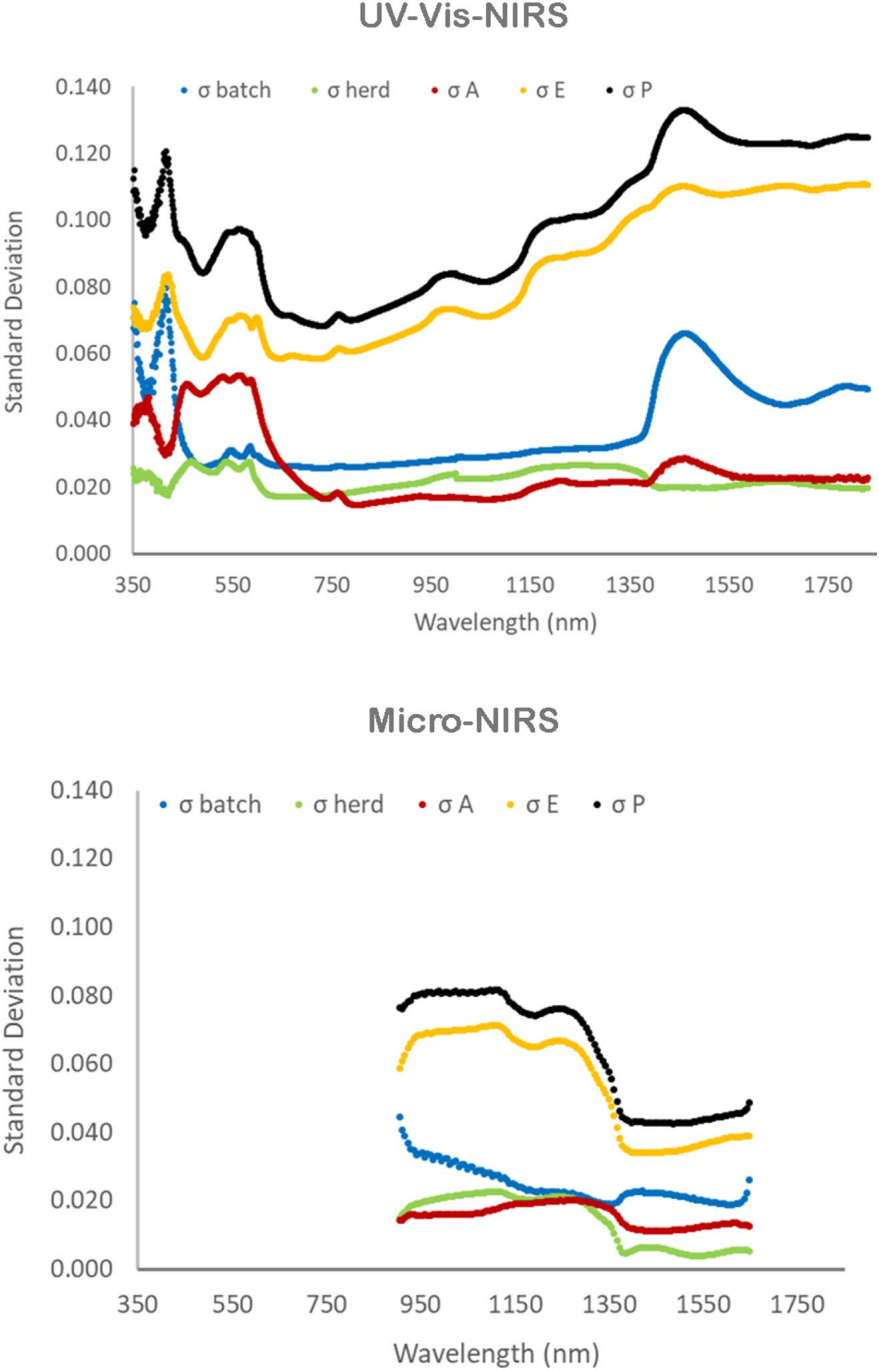
Standard deviations of the different sources of variation in absorbance. Phenotypic (σ_P_, black color), batch (σ_batch_, blue color), herd (σ_herd_, green color), additive genetic (σ_A_, red color), and residual (σ_E_, yellow color) standard deviations of absorbance of each individual wavelength of the spectra.

The proportions of the variances of the random factors included in the model (farm of rearing, batch/date of slaughter, animal additive genetic, and random residual) are reported in Fig. 2, and the amount of variation, in linear original scale of the trait (standard deviation), due to each source of variation is summarized in Fig. 3.

The figures show that the variability in absorbance due to the effect of batch/date of slaughter is particularly large for the wavelengths < 450 nm of the Vis–NIR spectrum (30–40% of total phenotypic variance), and for the near-infrared wavelengths recorded by both instruments in the spectral interval 1400–1600 nm (up to 25%). The variability due to the young bulls’ farm of rearing is much lower than that due to batch/date of slaughter, and approaches 5–10% of phenotypic variance only for absorbance at wavelengths 450–1400 nm for the UV–Vis-NIR spectrum, and 905–1400 nm also for the Micro-NIRS spectrum, excluding the first 8 wavelengths. It is worth noting that the variability due to batch/date of slaughter is proportionally much lower at wavelengths < 1400 of the spectra of both instruments.

The effects of animal additive genetics on the absorbance of electro-magnetic radiations are appreciable for all the wavelengths, but their size is highly dependent on the wavelength of the spectrum and also in part on the instrument used. The much larger effect of the animal’s genetics on the absorbance by the meat surface recorded with the UV–Vis-NIRS at wavelengths < 590 nm is worth noting. This effect gradually decreases over the remaining part of the visible interval (590–740 nm), and is thereafter—in the NIR—much smaller. Note also that genetics had a larger effect on the absorbance values recorded by the Micro-NIRS than those recorded by the UV–Vis-NIRS in the spectral interval common to the two instruments (Fig. 2). Conversely, the residual variance was much larger for the NIR than for the UV and visible wavelengths.

**Figure 4:**
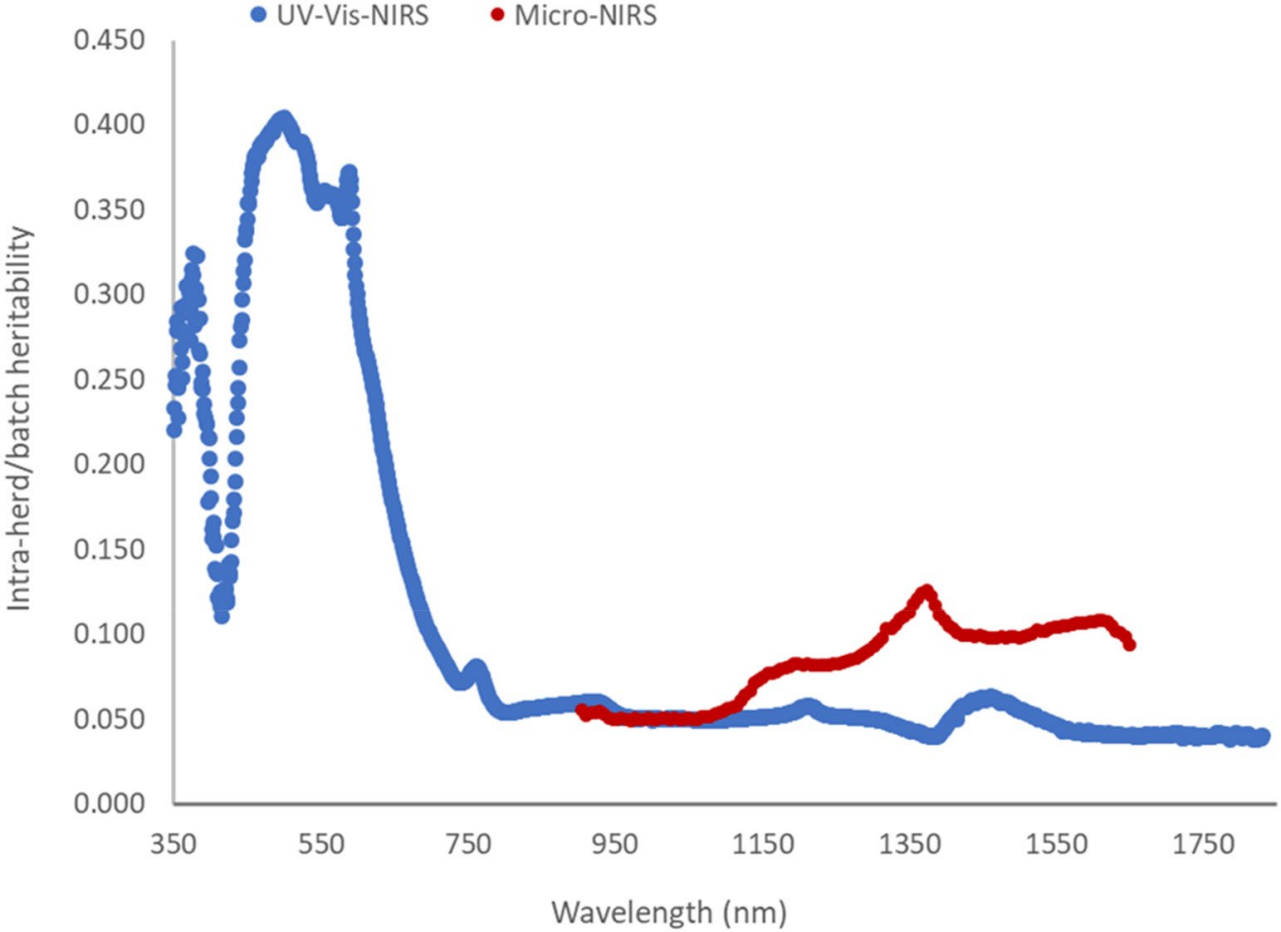
Intra-herd/batch heritability of absorbance of each individual wavelength of the spectra. Intra-herd/batch heritability of absorbance of each individual wavelength of the spectra taken on the 5–6th rib cross-sectional area of *longissimus thoracis* muscle.

Intra herd/batch heritabilities [additive genetic variance/(additive genetic + residual variances); i.e. after removing from the phenotypic variance the components due to batch/date of slaughter and farm of origin] are shown in Fig. 4 for each wavelength of the spectra of both spectrometers. Summarizing, the heritability of the absorbance of all wavelengths in the UV and visible portion of the UV–Vis-NIRS spectrum was always > 10%, whereas in the NIR portion it was generally < 10% (Fig. 4). The heritability of the absorbance recorded by the Micro-NIRS was greater than that of the corresponding portions of the UV–Vis-NIRS spectrum, particularly for wavelengths > 1400 nm (Fig. 4).

## Discussion

### The absorbance spectrum of beef meat

Many different spectrometers have been used for measuring the absorbance of electro-magnetic radiation in meat^1,23,32^. As well as near-infrared (NIR), ultra-violet^33,34^, visible^35^, and mid-infrared^33,36^ radiations have also been used.

Different institutions and authors subdivide the electro-magnetic radiation spectrum differently. It is commonly accepted that the various colors of visible light cover the interval 380–740 nm^37^, and that wavelengths < 380 nm belong to ultra-violet range, and those > 740 nm to the infrared range. The ISO (2007)^38^ classifies wavelengths 780–3000 nm as near-infrared (NIR), those between 3000 and 50,000 nm as mid-infrared (MIR), and > 50,000 nm far-infrared (FIR). However, the first infrared interval is often divided into two parts: < 1400 nm and > 1400 nm. Our results on the absorbance of infrared radiation by beef meat substantiates this subdivision as the average absorbance (Fig. 1)^27^, proportion (Fig. 2), and deviation of the different sources of variation in absorbance (Fig. 3), and, in part also its heritability (Fig. 4) are clearly different in these two portions. The first is often called near-infrared (NIR) in the narrow sense, the second the short-wavelength infrared (SWIR), although CIE (Commission Internationale de l´Eclairage, 2004)^39^ terms them infrared A and B (IR-A and IR-B), respectively, while the MIR interval is termed IR-C. To facilitate discussion of the results, we divided the entire spectrum of both instruments into ultra-violet, visible (different colors) and infrared radiations (IR-A and IR-B); Table 1summarizes the main results according to these intervals.

**Table 1:** Descriptive statistics and summary of major results according different spectral intervals of UV–Vis-NIRS and Micro-NIRS.

	Wave-lengths:		Absorbance		Average variance proportion (%):				Average heritability^b^
	Interval (nm)	Waves no	Mean	SD	Batch-date	Farm	Genetic	Residual	
**UV–Vis-NIRS**	350–1830	1481	1.165	0.495	14	6	8	72	0.105
Ultra-violet	350–380	30	1.223	0.021	30	5	18	47	0.279
Visible	380–740	360	0.990	0.392	15	7	20	58	0.255
Violet	380–450	70	1.556	0.147	30	4	14	52	0.206
Blue	450–485	35	1.104	0.108	10	9	31	50	0.382
Cyan	485–500	15	0.953	0.004	9	9	33	49	0.401
Green	500–565	65	1.145	0.131	9	8	31	52	0.377
Yellow	565–590	25	1.262	0.055	10	8	29	53	0.357
Orange	590–625	35	0.769	0.132	11	6	24	59	0.283
Red	625–740	115	0.538	0.029	14	6	10	70	0.130
Infra-red	740–1830	1091	1.220	0.518	14	5	4	77	0.050
IR-A, NIR^a^	740–1400	660	0.847	0.292	12	7	4	77	0.053
IR-B, SWIR^b^	1400–1830	431	1.793	0.082	18	3	4	75	0.046
**Micro-NIRS**									
Infra-red	905–1649	125	1.130	0.294	17	5	7	71	0.083
IR-A, NIR^a^	905–1397	83	0.962	0.215	13	7	6	74	0.074
IR-B, SWIR^b^	1403–1649	42	1.461	0.035	23	1	8	68	0.102

^a^Infra-red A or near-infrared (narrow sense); ^b^Infra-red B or short-wavelength infrared. The intra-herd-batch heritability is different for each wavelength (please see Fig. 4) and the ranges of standard error were 0.035–0.088 for UV–Vis-NIRS and 0.026–0.067 for micro-NIRS.

We are unaware of any study quantifying the major sources of variation in the absorbance of electromagnetic radiation by beef or other types of meat at individual wavelengths or at specific intervals of the spectrum. This being the first such study, the discussion cannot include comparisons with other published results.

### Variation in the absorbance of ultra-violet radiation by beef meat

Ultraviolet spectrometry has very seldom been studied in relation to food products, but interest is growing in the wake of new instrumentation, as reviewed by Martelo-Vidal and Vázquez^40^ and Power et al.^34^. A few studies have dealt with meat products^41^, and although the results are less promising compared with visible, NIR and MIR spectroscopy, they improve overall when combined with visible light (UV–Vis)^33,42^. The present study is the first to find batch/date of slaughter to be an important source of variation in ultraviolet absorbance (14% of total variance; Table 1), or at least in near ultraviolet absorption as our instrument does not measure absorbance of deep ultraviolet radiation. Animal genetics follows batch/date of slaughter in importance (18%), whereas farm of origin exerts only a modest effect. Consequently, the residual variance represented less than half the total phenotypic variance, i.e. the smallest proportion among all the spectral intervals obtained with UV–Vis-NIRS, while intra herd/batch heritability (28%) was one of the highest. In a previous research on the variability between two sides of the same carcass and among 5 locations on the surface of the same meat muscle sample^27^, we found both these within-animal sources of variation to be important for ultraviolet absorbance by beef meat.

### Variation in absorbance of visible light by beef meat

With regard to the absorbance—and reflectance—of visible light, the scientific literature contains a very large number of studies on meat, including beef^43^, which of course are most frequently aimed at characterizing meat color^44,45^. Our results showed absorbance by the cross-sectional surface of the *Longissimus thoracis* muscle to be very high for violet and green colors, intermediate for blue, cyan and yellow, and much lower for orange and red (Fig. 1). This is expected given that reflectance is the complement of absorbance and beef is a red meat. There is no information on the major sources of variation in the absorbance of meat at single wavelengths and individual colors of visible light. As Table 1 shows, violet color and ultraviolet radiation are very similarly affected by the major sources of variation. All the other colors are much less affected by batch/date of slaughter (9–14%, compared with 30% for violet and ultraviolet), slightly more by farm of origin (6–9%, vs 4–5%), and variably by animal genetics, which represented 29–33% of phenotypic variation in the case of blue, cyan, green, and yellow colors, 24% for orange, and only 10% for red (against 14% for violet color, 18% for ultraviolet radiations). As a result, residual individual variance represented 49–59% of the total variance for all colors except red (70%).

In our previous study on within-animal variation, we found that carcass side was an appreciable source of variation for violet, blue, cyan, green and yellow, but not for orange and red, and location on the muscle surface was a large source of variation, especially for violet and to a lesser extent yellow^27^.

To develop secondary prediction methods in meat products, absorbance of the visible spectrum was not used alone but usually in combination with ultraviolet (UV–Vis), as seen in the previous paragraph, or with NIR (UV–Vis-NIRS), as in this study.

### Variation in absorbance of infra-red radiation by beef meat

A large number of studies have used infrared absorbance spectra for predicting the physical, chemical or sanitary-hygienic properties of meats, and for authenticating or discriminating them^1–3^. The only available description of variability in meat absorbance is a plotting of all the individual spectra, their standard deviations, and the average absorbances of different groups of spectra across different wavelengths^7^.

Our results, illustrated in Figs. 1, 2, 3 and 4 and summarized in Table 1, clearly show that, regarding the UV–Vis-NIRS spectrum, batch/date of slaughter and farm of origin of the animals have a similar effect on the absorbance by beef of the infrared wavelengths as on the absorbance of the visible colors, except violet (much greater effect) and ultraviolet radiations (slightly smaller effect). Animal genetics is much less important, and the residual variation much more important. We can also see that, within the infrared waves, the first section (NIR or IR-A) differs from the second (SWIR or IR-B) in that the latter is more affected by batch/date (23 vs 13%), almost unaffected by farm of origin (1 vs 7%), similarly influenced by animal genetics (8 vs 6%), and slightly less affected by residual individual variation (68 vs 74%).

In a study on a different food matrix, cheese, we compared the predictive ability of different fractions of the UV–Vis-NIRS spectrum and found that the visible part tended to be slightly less accurate than the infrared segment for predicting chemical composition, similarly accurate for predicting texture and lightness, and much more accurate for predicting the other color traits^46^. Moreover, using the entire spectrum, combining visible and infrared fractions, never yielded more accurate results than using the best fraction.

Comparison of the two instruments provides new knowledge about the relationships between their technical features and the results obtained. First, it is clear from Fig. 1 that the patterns of absorbance recorded by the two instruments in the common part of the first infrared fraction (NIR, IR-A) are very similar, whereas in second fraction (SWIR, IR-B) the Micro-NIRS yielded lower and less variable values than the UV–Vis-NIRS, and this lower variability regarded all the divergences produced by the major sources of variation (Fig. 3), although in relative terms it concerned the farm and residual effects more than batch/date of slaughter and additive genetics (Table 1). Note that, using this same dataset, the predictions of beef quality traits based on UV–Vis-NIRS spectra were characterized by a greater calibration accuracy than those based on Micro-NIRS spectra^27^, but the advantage decreased moving from calibration to cross-validation and tended to disappear with external validation. Using a different database, predictions of the mineral contents of beef assessed by external validation were even more accurate with the Micro-NIRS than with the UV–Vis-NIRS spectra^8^. Research on the prediction ability of the two infrared fractions with different instruments is lacking. Comparison of five different sources of information for authenticating milk and cheese, including NIRS and FTIRS, revealed that a high number of data-points per sample favored high accuracy of calibration equations, but did not influence the results when externally validated^47^.

### Heritability of absorbance values for different ultraviolet, visible and infrared radiations, and possible use for genetically improving beef meat

We are unaware of any published study on the heritability of the absorbance of ultraviolet, visible and infrared radiations by beef meat or meat from other species.

Some research has, however, been done on the heritability of the absorbance of infrared radiations by milk, although the spectra were generally not obtained by ultraviolet, visible and near infrared spectrometers, but rather by Fourier transform infrared (FTIR) spectrometers combining the SWIR IR-B fraction characterized by long waves (2000–3000 nm; corresponding to 5000–3333 waves/cm), with MIR (3000–8000 nm; 3333–1250 waves/cm) and LIR (8000–10,760 nm; 1250–833 waves/cm)^48^.

Earlier genetic analyses of milk were not carried out on the absorbance of milk at every individual wavelength, but on their principal components in order to reduce the size of the database^49^. We directly estimated the heritability of every wavelength of the milk FTIR spectrum in Brown Swiss cows^48^ and obtained values between 5 and 10% for the SWIR IR-B fraction, similar to those found here for the SWIR IR-B fraction of the beef spectrum. In a recent study^30^, we confirmed similar heritability values for the SWIR IR-B fraction of the milk spectrum in Holstein Friesian cows, and found that heritability was affected by parity and, especially, the cow’s lactation stage^29^, found an effect of herd on the absorbance of IR-B radiation (2000–2500 nm) by milk similar to or greater than in the present study, and a large genetic effect, more similar to that recorded for the absorbance of visible radiations by beef.

In the plant kingdom, Hein and Chaix^50^ found a broad-sense heritability of absorbance of infrared radiations (1111–2500 nm) by wood ranging from 0 to over 50% according to the individual wavelength, and considered NIRS a promising tool in breeding programs of forest tree species for several wood traits. Here, too, the average heritability was greater for IR-B (~ 20%) than for IR-A (~ 10%) radiations.

In the present study, we found that absorbance of electromagnetic radiation by beef meat has a much higher heritability at individual wavelengths in the ultraviolet and visible fraction of the UV–Vis-NIRS spectrum than those in the infrared fractions (Fig. 4). The highest average heritability was for absorbance of blue, cyan, green and yellow radiations, followed by orange and ultraviolet (Table 1). The red wavelengths had a much lower heritability of absorbance than the other colors, but a higher heritability than the infrared radiations.

The heritabilities of color traits measured in CIE Lab values using this same dataset was 13–14% for redness, yellowness, and derived chroma and hue traits^25^, i.e. very similar to the average heritability value observed here for the 115 wavelengths in the red region (Table 1), but much lower than average value for the 25 wavelengths typical of yellow color. Heritability of the lightness trait (31%) was much higher, and was closer to the average heritability value (26%) of all 360 visible wavelengths recorded in the UV–Vis-NIRS spectrum. On the other side, lightness depends on all the visible colors. We found an almost identical heritability (32%) for beef lightness in a previous research within a different survey^51^, whereas the results were much more variable for the other color traits (14% for yellowness to 66% for hue angle). Similar values for beef color traits were also found in the Australian environment^52,53^. A subjectively evaluated beef color score was much less heritable (6%) than the measured traits^54^.

**Figure 5:**
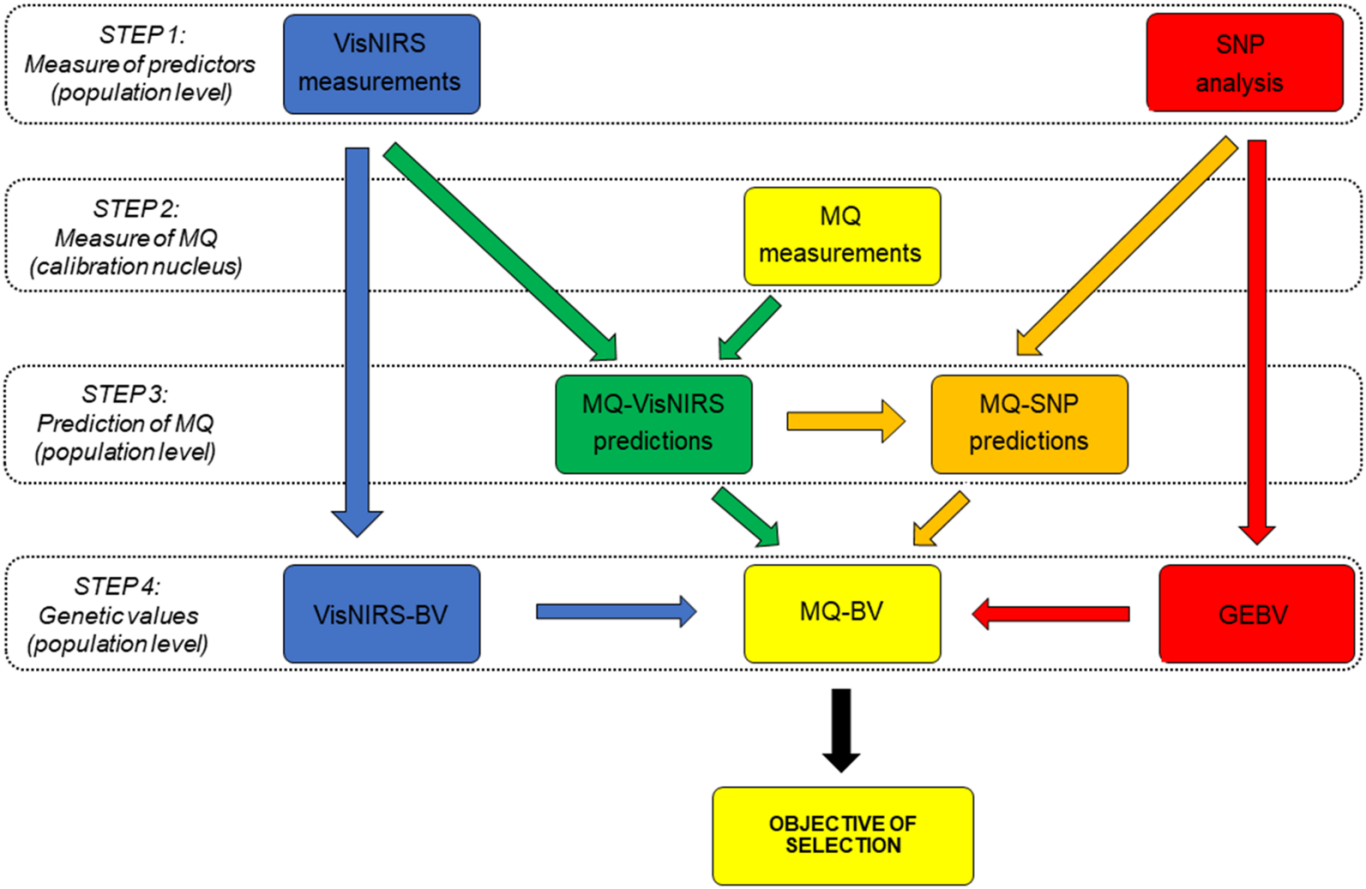
Schematic approaches for improvement of meat quality (MQ). Schematic approaches for improvement of MQ through: direct phenotypic selection from reference meat quality measurements at nucleus level (yellow pathway); indirect genetic selection (green pathway); direct genetic selection (blue pathway); indirect genomic selection (orange pathway) and direct genomic selection (red pathway).

Figure 5 illustrates alternative pathways for the genetic improvement of meat quality in a beef cattle population. As direct phenotypic selection (yellow pathway) at the population level is unfeasible because of cost and complexity, the most obvious use of beef spectra is to take an “indirect genetic selection” approach (in green in Fig. 5). Improvement in meat quality is sought through spectroscopic prediction of phenotypes at the population level using appropriate chemometric approaches (within a calibration nucleus within the population), and then by estimating the breeding value of candidates for the predicted meat quality traits. The heritability of predicted traits, and especially the genetic correlations between the reference and predicted meat quality traits, are obviously the most important indicators of the feasibility and efficiency of this indirect phenotypic selection pathway, and they differ for different traits^10^.

Genetic comparisons of reference and predicted traits in dairy animals have been carried out, often with good results, for several traits: milk fatty acid profiles^11^, milk technological properties^55^, and enteric methane emission traits^56^. Unlike dairy populations, comparison of the genetic parameters of quality traits measured by gold-standard methods with those predicted by spectroscopic methods in beef populations have been carried out only in a previous research by our group^10^. The results were promising, but the instrument used there was a bench-top laboratory NIR spectrometer, which required beef samples to be taken, transported to the laboratory, and processed before the spectra could be retrieved. In the present research, we used portable instruments, which can take spectra directly on the meat surface in the abattoir or meat processing plant without the need to collect and process samples^27^. Here, too, the results of analysis of the genetic correlations between the reference methods and spectroscopic predictions are favorable for both instruments^28^.

It is worth noting that the appreciable heritability of the absorbance of many individual wavelengths by beef meat opens new possibilities for the genetic improvement of beef quality traits. A different “direct genetic selection” approach can be taken (blue pathway in Fig. 5), where we hypothesized that “VisNIRS-breeding values”, which are dependent only on Vis-NIRS measurements and without any information on meat quality measurements, could be used for predicting meat quality breeding values taking into consideration the genetic correlations (currently not known) between the spectral wavelengths and the meat quality traits.

Dagnachew et al.^57^ showed that using genetic breeding values of principal components of FTIR milk spectra to directly predict the breeding values of dairy goats for milk composition (direct genetic selection) is more efficient than using milk spectra for indirect genetic prediction of milk composition. An interesting future research line would be to apply similar approaches to meat animals.

On the other side, in the genomic era, beside the use of genomic information to predict breeding values for meat quality traits (direct genomic selection approach, the red pathway in Fig. 5), it is essential to research the genome-wide foundations of meat absorbance properties (the orange pathway in Fig. 5), as has been done for milk spectra^31^. Additionally, Compared with dairy populations, genomic selection in beef populations suffers from a much lower incidence of artificial insemination and the high cost of phenotypic measurement for system calibration, especially in the case of meat quality traits. In this context, meat spectra could play a double role. The simplest one would consist in predicting meat quality phenotypes of the genomic calibration nucleus; the alternative would be to include in genomic selection models the markers of absorbance wavelengths genetically associated with meat quality^58^.

Every section of the spectrum, and in part also every individual wavelength, is affected to different degrees and proportions by the animals’ batch/date of slaughter, farm of origin, additive genetics, and the residual variation. The heritabilities of absorbance are much greater for wavelengths in the ultraviolet fraction and the violet, blue, cyan, green, yellow, and orange regions; they are lower for those responsible for red color, and very modest for those in the infrared fractions of the spectrum. The heritabilities of absorbance properties of meat make it possible to estimate the breeding values of animals for these phenotypes, or for phenotypes predicted from the spectra, and to use this information for improving meat quality aptitude at the population level. Moreover, these results motivate the study of meat quality at the genome-wide level and testing for calibration of possible genomic selection schemes.

Lastly, we found that the miniaturized Micro-NIRS, despite its much shorter and less clearly defined spectrum, produced absorbance values of meat with a larger heritability than the reference instrument with its much wider and more clearly defined spectrum.

## Methods

### Animals

Spectra were obtained from 1185 Piemontese young bulls slaughtered at the same commercial abattoir. The young bulls were progeny of 200 A.I. purebred sires and 1150 dams, all registered in the Italian Piemontese Herd Book and selected mainly for growth rate, muscularity and direct and maternal ease of calving^59,60^.

The animals were fattened on 93 commercial farms representative of the beef production systems in the Piemonte region (north-west Italy). The farming systems, feeding regimes, fattening conditions and slaughter performances of the young bulls are described in detail by Savoia et al.^24^. Live animals were not handled during this experiment and data were collected after slaughtering of animals in a commercial abattoir. All procedures were performed in accordance with the relevant guidelines and regulations. At slaughter, the young bulls had an average carcass weight of 438.3 (± 45.8) kg, and an average age of 538 (± 62) days, giving an average daily carcass gain of 0.822 (± 0.109) kg/day. The average carcass conformation score (using the SEUROP classification system with each category divided into three subclasses giving a scale of 1–18 points) was 14.7, corresponding to an average evaluation close to “E+” in the EU linear grading system. The average ribeye area measured at the 5th rib was 92 cm^2^ (± 16).

### Spectra collection

Spectra collection and the instruments’ technical characteristics are described in detail by Savoia et al.^27^ and summarized in Table 2.

**Table 2:** Main characteristics of the spectrometers used for predicting quality traits of beef and data used.

	UV–Vis-NIRS	Micro-NIRS
**Instrument**		
Denomination	LabSpec 2500	Micro NIR Pro
Type	Portable	Hand-held
Spectrometer weight	5600 g	60 g
Power source	Internal battery or cable	USB 2.0, (480 Mbps)
Light source	Halogen	Two vacuum tungsten lamps
Light detection	External probe	Internal
External probe weight	654 g	–
Spectra storage	Internal	External PC or tablet
**Spectrum acquisition**		
Sample preparation	None	None
Waves range	350–1830 nm	905–1649 nm
Data point interval	1 nm	6 nm
Replicates per spectrum	3	3
Spectra collected per sample	5	5
**Spectrum pretreatments:**		
Absorbance calculation^a^	A = log(1/R)	A = log(1/R)
Average spectrum per animal	Yes	Yes
Mathematical pre-treatment	Centering, standardization	Centering, standardization
Editing on the basis of	Mahalanobis distance	Mahalanobis distance
**Data available, N**		
Data point per spectrum	1481	125
Animals/average spectra	1181	1185
Individual spectra	17,715	17,775
Absorbance measurements	26,235,919	2,221,875
Pedigree animals	13,102	13,182

Briefly, the spectra were acquired with the following spectrometers:

- UV–Vis-NIRS: LabSpec 2500 (ASD Inc., Boulder, CO, USA); dimensions 12.7 × 36.8 × 29.2 cm, weight 5600 g; spectra are collected with a probe (26 × 10 × 5 cm; 654 g) connected to the instrument with an optical fiber; the instrument’s spectral range is in the near-ultraviolet, visible and near-infrared sections of electromagnetic radiation (wavelengths 350–1830 nm); measurements are taken every 1 nm producing 1481 data points per sample;
- Micro-NIRS: Micro NIR Pro (JDSU, San Jose, CA, USA); dimensions 4.5 × 4.4 × 4.0 cm, weight 60 g; spectra are collected directly by the instrument, which should be connected to a lap-top or tablet via a USB cable; the instrument’s spectral range is in the near-infrared region (wavelengths 905–1649 nm); measurements are taken every 6 nm producing 125 data points per sample.

The right side of each carcass was divided into two quarters (pistol cut) in the abattoir the day after slaughter (about 24 h post-mortem). The spectra were collected on the cross-sectional surface of the *Longissimus thoracis* muscle between the 5th and 6th ribs using the scanning head of the fiber-optic contact probe (diameter 10 mm) of the UV–Vis-NIRS or by applying the Micro-NIRS directly to the surface of the muscle. Five spectra were acquired with each instrument from different positions on the cut surface of the same muscle, each one an average of three replicates in the same position.

### Statistical analyses

#### Spectral data editing

The spectral data were edited and processed according to the model described in detail in Savoia et al.^27^ In brief, records with errors (e.g. individually identified spectra not matching the reference samples) and outlier spectra identified by Mahalanobis distance were discarded from the original spectral datasets obtained with the two spectrometers. The spectral data were centered and standardized prior to genetic analyses. The averages and standard deviations of the absorbances registered by the two instruments on the meat surface at every individual wavelength of their respective spectra obtained in the previous study are shown in Fig. 1.

#### Estimates of (co)variance components and genetic parameters

(Co)variance components were estimated by REML procedures using the VCE software version 6.0^61^. The absorbance values of each of the wavelengths of the two instruments were analyzed separately (1481 analyses for UV–Vis-NIRS, 125 for Micro-NIRS). The general model in matrix notation was:

$$\boldsymbol{y}=\boldsymbol{W}1\boldsymbol{c}+\boldsymbol{W}2\boldsymbol{q}+\boldsymbol{Z}\boldsymbol{u}+\boldsymbol{e},$$

where **y** contains the absorbance observations for a given instrument/wavelength, **c** is the vector of random herd effects (93 levels), **q** is the vector of the random effect of batch/day of slaughter (115 levels), **u** is the vector of animal additive genetic effects, **e** is the vector of random residual effects, and **W1**, **W2** and **Z** are the incidence matrices of appropriate dimensions. The random effects of herd (**c**), batch/day of slaughter (**q**), additive genetic (**u**) and residual (**e**) were assumed to be normally distributed: $\boldsymbol{c}\sim\,N\left(0,\boldsymbol{I}{{\sigma}}_{\text{c}}^{2}\right)$, $\boldsymbol{q}\sim\,N(0,\boldsymbol{I}{{\sigma}}_{\text{q}}^{2}$),$\boldsymbol{u}\sim\,N(0,\mathbf{A}{{\sigma}}_{\text{u}}^{2})$ and $\boldsymbol{e}\sim\,N\left(0,\mathbf{I}{{\sigma}}_{\text{e}}^{2}\right),$ where ${{\sigma }}_{\text{c}}^{2}{,{\sigma}}_{\text{q}}^{2},{{\sigma }}_{\text{u}}^{2}$, and ${{\sigma }}_{\text{e}}^{2}$ are the variance components for the herd, batch/day of slaughter, additive genetic, and residual effects, respectively; **A** represents the numerator relationship matrix between individuals (Wright, 1922; the pedigree file contained data on 6031 animals), and **I** is an identity matrix. A minimum cell size of three observations was required for both batch and farm effects. Additive relationships were computed from a pedigree file that included all phenotyped animals and their known ancestors.

Intra-farm/batch heritability was computed as ${h}^{2}={\sigma }_{a}^{2}/\left({\sigma }_{a}^{2}+{\sigma }_{e}^{2}\right)$, where σ^2^_a_ is the additive genetic variance, and σ^2^_e_ is the residual variance.

## Data Availability

The datasets generated and/or analysed during the current study are available from the corresponding author on reasonable request.
